# Challenges in isolating silica particles from organic food matrices with microwave-assisted acidic digestion

**DOI:** 10.1007/s00216-019-01964-2

**Published:** 2019-06-21

**Authors:** Otmar Geiss, Ivana Bianchi, Chiara Senaldi, Josefa Barrero

**Affiliations:** Directorate F Health, Consumers and Reference Materials, Joint Research Centre, European Commission, Via E. Fermi, 2749, 21027 Ispra, VA Italy

**Keywords:** Silica nanoparticles, E551, Matrix removal, AF_4_, ICP-MS, DLS, Microwave digestion

## Abstract

Synthetic amorphous silica is widely used in food processing as a food additive (E551) due to its properties as a flavour carrier and anti-caking agent. The direct measurement of E551 suspended or embedded in complex matrices is difficult without prior removal of the matrix components. The isolation of nanoparticles from the matrix is hence the first step towards their comprehensive characterization. Due to its complexity, matrix removal is frequently not trivial and may cause modification of the number-size distribution of the silica particles. The isolation of engineered silica nanoparticles by removal of the matrix with microwave-assisted acidic digestion is demonstrated methodologically using both monodisperse (size standards) and polydisperse (E551) particles spiked into ultrapure water and tomato sauce. For the characterization of the isolated nanoparticles, asymmetric field flow fractionation (AF_4_) coupled to multi-angle laser light scattering (MALS) and inductively coupled plasma mass spectrometry (ICP-MS) were chosen. The combination of ICP-MS and ultracentrifugation allowed for the rapid and reliable measurement of the dissolved fraction of SiO_2_. The results show that microwave-assisted acidic digestion partially dissolves silica nanoparticles. Moreover, the digestion conditions, in particular the low pH value, lead to strong agglomeration of the particles. A complete deagglomeration is not achieved, even when exposing the suspension to elevated sonication doses. The consequence of these two findings is a size distribution of particles after acidic digestion that is different from the original distribution before digestion. This result may have an impact on the evaluation of whether the material is a nanomaterial according to the recommended definition of the European Commission.

**Electronic supplementary material:**

The online version of this article (10.1007/s00216-019-01964-2) contains supplementary material, which is available to authorized users.

**Figure:**
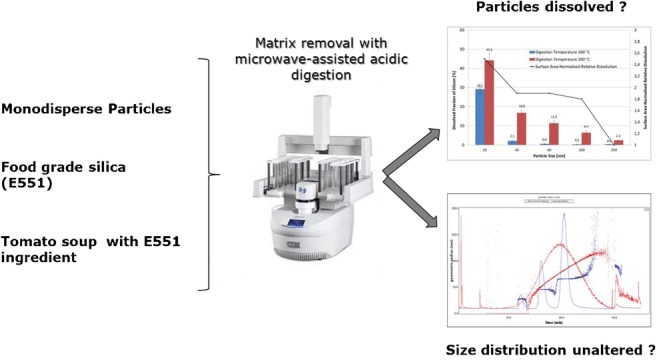
Graphical abstract

## Introduction

The use of nanoparticles (NPs), including silica nanoparticles (SiO_2_-NPs), has increased intensely in the last years thanks to the technical benefits they offer [1, 2]. In addition to the wide range of non-food application fields [3], synthetic amorphous silica (SAS) is being used in food processing as a food additive (E551) due to its property as a flavour carrier and anti-caking agent [4, 5]. Silicon dioxide (E551) is an authorized food additive in the EU and must be labelled according to the provision of Regulation (EU) No 1169/2011 [6] on food information to consumers. According to Article 18(3) of this regulation, all ingredients in the form of engineered nanomaterials shall be clearly indicated in the list of ingredients of prepacked foods marketed in the EU. To implement and enforce such a regulation, analytical methods to detect, characterize and quantify engineered nanoparticles (ENPs) are required.

Engineered nanoparticles (ENPs) in consumer products, such as cosmetics or foodstuffs, are usually suspended or embedded in complex matrices and can therefore not be directly measured without prior removal of the matrix components. Therefore, the isolation of nanoparticles from the matrix is the first step towards their comprehensive characterization. Nevertheless, due to the matrix complexity and the strong interactions between some of its components and the ENPs, matrix removal is not trivial [7], and many of the reported methods can modify the ENPs in terms of mass concentration and number-size distribution. To date, no universally applicable isolation and measurement method is available, and any procedure must be carefully tailored to the matrix/material system. Several methods are described to release NPs from a sample, such as matrix destruction, extraction and/or purification. Chemical destruction of the organic components of the matrix can be achieved through acidic or alkaline digestion [2, 8–10] as well as through peroxy (Fenton) reaction [11, 12]. An alternative method for the separation of chemically sensitive materials from the matrix is enzymatic digestion, in which α-amylase for carbohydrate degradation and protease for protein degradation are added [9, 13, 14].

**Fig. 1:**
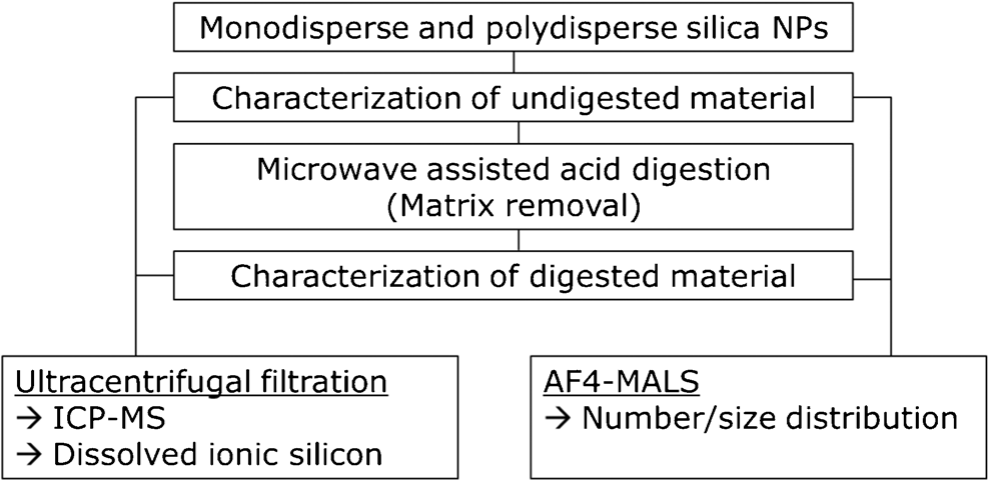
Schematic of experimental procedures

This study aims at systematically assessing whether microwave-assisted acidic digestion is a suitable method for the separation of SiO_2_ nanoparticles from matrices. The isolation of engineered silica nanoparticles by removal of the matrix with microwave-assisted acidic digestion is demonstrated methodologically using monodisperse and polydisperse (E551) particles spiked in ultrapure water and tomato sauce. For the characterization of the isolated nanoparticles, asymmetric field flow fractionation (AF_4_) coupled to multi-angle laser light scattering (MALS) and inductively coupled plasma mass spectrometry (ICP-MS) were chosen. ICP-MS analysis rapidly provides reliable information on the dissolved fraction of SiO_2_ as it corresponds to the difference in Si-28 mass concentrations found before and after centrifugal filtration. The particle size distribution is assessed by analysing the silica extracts with AF_4_-MALS. Figure 1 schematically depicts the experimental procedures described in this study.

## Materials and methods

### Chemicals

The ultrapure water used in this work (18.2 MΩ.cm at 25 °C and TOC ≤ 5 ppb) was obtained from a Milli-Q Integral 3 System (Millipore, Billerica USA). For acidic digestion, 67–69% traceSELECT™ ultra HNO_3_ (Honeywell International, USA, Product Code 02650) and 30% H_2_O_2_ (Sigma-Aldrich, St. Louis, USA, Product Code 95321) solutions were used. Yttrium (Product Code 01357) and silicon standards (Product Code 08729) for ICP-MS, as well as ammonium carbonate (Product Code 379999), were purchased from Sigma-Aldrich (Sigma-Aldrich Corp., St. Louis, USA).

### Silica nanoparticles (monodisperse standard suspensions)

Size standards of SiO_2_ nanoparticles in water with nominal diameters of 20, 40, 60, 80, 100 and 150 nm (certified modal diameters and expanded uncertainties are listed in Table 1) were purchased from MSP Corporation (Shoreview, MN, USA). Stock suspensions were stored in the dark following the supplier’s recommendations. Dilutions and working suspensions from the stock materials were freshly prepared each week. Before use, the concentrations of the standards, reported in Table 1, were determined/verified with ICP-MS, while the sizes were confirmed with dynamic light scattering (DLS).

**Table 1:** Certified modal diameters with their expanded uncertainties and mass concentrations of monodisperse silica standards used in this work

Certified modal diameter (nm)	Determined mass concentration (mg mL^−1^)
20.2 ± 0.5	7
39.1 ± 0.9	56
61.7 ± 1.6	34
78.6 ± 1.9	16
98 ± 2.6	37
154 ± 4	76

### Food-grade synthetic amorphous silica E551 (SAS)

The polydisperse food-grade synthetic amorphous silica (SAS) used in this study for the spiking experiments was Cab-o-Sil EH-5F manufactured by the Cabot Corporation (Boston, USA). Cab-o-Sil EH-5F is a food-grade hydrophilic fumed silica used for a wide variety of applications—including as a food additive (E551), where it acts as a flavour carrier and anti-settling, thickening and anti-caking agent. Cab-o-Sil EH-5F is a high-purity food additive in compliance with applicable EU and US food additive regulations.

### Tomato sauce used as a real matrix for spiking experiments

The tomato sauce used in this study for spiking experiments was purchased at a local market in Italy with the commercial name “Carrefour Passata di pomodoro”.

### Elemental analysis of silica nanoparticles by ICP-MS

The elemental analysis was performed using a Perkin Elmer NexIon 300D quadrupole ICP-MS (Perkin Elmer, Waltham, MA, USA) equipped with an SC fast peristaltic pump. The most notable/significant interference on the major silicon isotope (Si-28) is ^12^C^16^O^+^ followed by ^14^N_2_^+^ [15]. Studies conducted in the past showed that the highest contributions to the Si background were coming from the sample introduction system and the lab-ware used for the preparation of samples [15–17]. For this reason, a PFA-ST nebulizer, a PFA 7-mm Baffle cyclonic chamber and a ceramic T-torch, complete with a PFA/Sapphire injector, were used in this study and all samples/solutions were prepared using polymeric instead of glass lab-ware. A number of previous studies also demonstrated that the polyatomic interferences could effectively be reduced using ammonia as the reaction gas [17, 18]. Following these indications, the system was operated in dynamic reaction cell mode (DRC) at an ammonia flow of 0.8 mL min^−1^, with an RPq of 0.7, a monitoring isotope of *m*/*z* 28 for silicon, a dwell time of 50 ms and an integration time of 1 s.

Quantifications were done against yttrium (*m*/*z* 89), used as an internal standard to compensate for changes in the signal intensity caused by changes in the spray formation. For each set of samples, a new 7-point ionic silicon calibration curve ranging from 5 to 500 ppb was created and all samples were analysed in triplicate. Under these conditions, the limits of detection and of quantification were determined according to formulas (1) and (2):

1 $$ \mathrm{Limit}\ \mathrm{of}\ \mathrm{detection}\ \left(\mathrm{LoD}\right)=3\ast \mathrm{sd}\ \left(\mathrm{standard}\ \mathrm{deviation}\ \mathrm{of}\ 6\ \mathrm{replicates}\ \mathrm{of}\ \mathrm{the}\ \mathrm{lowest}\ \mathrm{point}\ \mathrm{in}\ \mathrm{the}\ \mathrm{calibration}\ \mathrm{curve}\right)/\mathrm{slope}\ \mathrm{of}\ \mathrm{the}\ \mathrm{calibration}\ \mathrm{curve} $$

2 $$ \mathrm{Limit}\ \mathrm{of}\ \mathrm{quantification}\ \left(\mathrm{LoQ}\right)=10\ast \mathrm{sd}\ \left(\mathrm{standard}\ \mathrm{deviation}\ \mathrm{of}\ 6\ \mathrm{replicates}\ \mathrm{of}\ \mathrm{the}\ \mathrm{lowest}\ \mathrm{point}\ \mathrm{in}\ \mathrm{the}\ \mathrm{calibration}\ \mathrm{curve}\right)/\mathrm{slope}\ \mathrm{of}\ \mathrm{the}\ \mathrm{calibration}\ \mathrm{curve} $$

The limit of quantification was 1.7 ppb which is in good agreement with the findings of Aureli and co-workers [17], who determined the limit of detection under the same conditions.

### Impact of acidic digestion on the dissolution of particles and the formation of ionic silicon

Among the principal objectives of the current work was to assess whether the relatively aggressive acidic microwave-assisted digestion procedure results in the complete or partial dissolution of the silica particles. This was done by digesting particles of known concentration and size, filtering the digested solution through nanoparticle-retentive centrifugal filters and analysing the presence of silicon in the filtrate. Each of these steps required optimization. The optimization procedures are described in the following.

#### Optimization of the centrifugal filtration step—release of ionic silicon from filter membranes

In the procedure proposed in this study, the digested and diluted solution is filtered to separate silica particles from the ionic silicon potentially released/dissolved during the digestion procedure. Amicon Ultra-4 centrifugal filters (Merck Millipore, Cork, Ireland, product code UFC800324) with a nominal molecular weight limit of 3 kDa, corresponding to a pore size of approximately 1 nm [19], were used for this purpose. Before use, the centrifugal filters were tested for their release of ionic silicon from the cellulose membrane. For this purpose, 4 mL of ultrapure water was centrifuged at 4000 rpm for 50 min at room temperature, and the content of ionic silicon in the filtrate was subsequently analysed with ICP-MS.

#### Nanoparticle retention efficacy of the filtering membrane

The retention efficacy of the filtering membrane was assessed by filtering suspensions of SiO_2_-nanoparticle standards (20 to 150 nm, each at 200 ppb in 1% HNO_3_) through centrifuge filters, which retain particles down to a size of 1 nm while not retaining the ionic silicon, under the conditions described in the section “Optimization of the centrifugal filtration step—release of ionic silicon from filter membranes”. The amount of silicon in the filtrate was then measured with ICP-MS, and the retention efficacy was calculated by dividing the concentration of silicon in the filtrate by that in the feed suspension. A blank sample (4 mL of 1% HNO_3_) was filtered and analysed in parallel for quality control purposes. For these tests, the filters were washed once with 1% nitric acid before use.

#### Microwave acidic digestion—optimization of conditions

As unnecessarily aggressive digestion conditions could enhance the dissolution of silica nanoparticles, in this set of experiments, the minimum conditions—in terms of temperature and time—necessary to digest the sample matrix were determined.

A CEM SP-D 80 microwave digestion system (CEM Corporation, NC, USA) was used in this study. The initial digestion conditions were taken from a pre-set method for the digestion of tomato sauce. A mixture of 2 mL tomato sauce, 5 mL concentrated HNO_3_ and 1 mL 30% H_2_O_2_ underwent digestion under the following conditions: target temperature 200 °C, ramp time 5 min, hold time 4 min and maximum power 300 W. As alternatives to these standard conditions, lower digestion temperatures (180 °C, 160 °C, 150 °C, 120 °C and 100 °C) and/or shorter digestion time periods (4, 3, 2 and 1 min) were tested. Digestion was considered complete when the digested solution was transparent to the naked eye. All digestion vessels were equipped with Teflon™ liners.

#### Correlation between nanoparticle size and silica dissolution at two target digestion temperatures (160 °C and 200 °C)

Particle suspensions of each available nominal size (20, 40, 60, 80, 100 and 150 nm) were directly prepared in the microwave digestion tubes equipped with Teflon™ inserts. The aqueous particle suspensions were prepared in such a way that the digested and diluted suspensions (ready for ICP-MS analysis) yielded 400 ppb each. A blank sample (2 mL of ultrapure water) and a control sample containing ultrapure water spiked with ionic silicon (200 ppb solution after digestion and dilution) were also prepared for quality control purposes. Digestion mixtures were completed by adding 5 mL of 69% HNO_3_ and 1 mL of 30% hydrogen peroxide to each tube. After 10 min of equilibration at room temperature, the samples were digested as described under the section “Microwave acidic digestion—optimization of conditions”, at two different target temperatures (160 °C and 200 °C). After cooling, the acidic samples were transferred from the digestion tubes into 100-mL volumetric flasks and brought to volume with ultrapure water. This reduced the concentration of HNO_3_ from 69% to approximately 3.5%, making the mixture suitable for both ICP-MS analysis and filtration through the Millipore Amicon filters. Yttrium, as an internal standard, was added at a concentration of 200 ppb to the ready-to-measure suspensions. The suspensions were then analysed with ICP-MS for the total content of Si-28. To determine the ionic content, 4 mL of the digested and diluted suspensions was added to an Amicon® Ultra-4 centrifugal filter device (Millipore, USA) and centrifuged for 50 min at 4000 rpm (Multifuge 3 S-R, Heraeus, Hanau, Germany). The filtrate was analysed with ICP-MS.

#### Solubility of polydisperse food-grade (E551) fumed silica under acidic digestion conditions

In all preceding experiments, monodisperse silica nanoparticles were used to assess the dissolution of silica nanoparticles in a systematic way. In this test, however, a polydisperse food-grade E551 was suspended and digested, and the dissolution was assessed. A suspension of 1000 ppm was prepared in ultrapure water, ultrasound probe sonicated (10 min at 24 W, 14,400 J) and then filtered through a 0.45-μm PTFE syringe disc filter. An aliquot of 100 μL of this suspension was transferred into a 100-mL volumetric flask, brought to volume with 1% HNO_3_ and analysed with ICP-MS. A fraction of 4 mL of the suspension was filtered through Amicon® filters and analysed by ICP-MS for the determination of the ionic silicon *before* microwave-assisted acidic digestion. In addition, 100 μL of the 1000 ppm suspension was added to a digestion tube with the Teflon™ insert equipped, along with 1.9 mL ultrapure water, 5 mL of 69% nitric acid and 1 mL of 30% hydrogen peroxide. After 10 min of equilibration, the suspension was digested as described in the section “Correlation between nanoparticle size and silica dissolution at two target digestion temperatures (160 °C and 200 °C)”. The digested suspension was probe sonicated (10 min at 24 W, 14,400 J) and, after a 1:100 dilution, analysed with ICP-MS. For the determination of ionic silicon, 4 mL of the suspension was filtered through Amicon filters and analysed by ICP-MS. Two replicates were performed (*n* = 3).

### Impact of acidic digestion on the nanoparticle size distribution

This second block of experiments was aimed at assessing whether acid microwave digestion of silica nanoparticles has an impact on their size distribution. This work was primarily based on the uses of asymmetric flow field flow fractionation (AF_4_) coupled to a multi-angle light scattering detector (MALS) and dynamic light scattering (DLS).

#### Dynamic light scattering (DLS)

A Zetasizer Nano-ZS (Malvern Panalytical Ltd., Malvern, UK) was used to perform dynamic light scattering measurements. All measurements were done in triplicate immediately after preparation at 25 °C, using disposable cuvettes. The DLS settings included the automatic optimization of the measurement conditions (measurement position/depth and attenuator).

#### Asymmetric flow field flow fractionation (AF_4_)-MALS

The AF_4_ used for the separation and analysis of the SiO_2_ nanoparticles included an Eclipse Dualtec separation system from Wyatt (Wyatt Technology Europe GmbH, Dernbach, Germany) and an Agilent 1260 Infinity high-performance liquid chromatograph equipped with a degasser (G1322A), an isocratic pump (G1310B), an autosampler (G1329B) and a multi-wavelength detector (G1365C), all from Agilent Technologies (Agilent Technologies, Santa Clara, USA). A Dawn 8+ Heleos II multi-angle laser light scattering (MALS) detector operating with a 658-nm laser (Wyatt Technology Europe) was coupled to the fractionation system. A 90° angle was used to monitor the signal during analysis. Regenerated cellulose (10 kDa) membranes were used in the Eclipse SC separation channel (153 mm length). The spacer height was 350 μm. The temperature of the channel was kept constant at 25 °C. The eluent was a 0.25-mM ammonium carbonate solution (pH 9), prepared freshly every day. The data acquired with the MALS detector were processed using the ASTRA® 6.1 software package (Wyatt Technology Europe). The flow programme and cross-flow (x-flow) settings are included in Table 2. The detector flow was set at 0.5 mL min^−1^, the injection volume was 50 μL and the focus flow was 1.0 mL min^−1^.

**Table 2:** AF_4_ flow programme and x-flow setting

Time [min]	Description	Cross-flow [mL min^−1^]
0–2	Elution	1
2–3	Focus	
3–6	Focus + inject	
9–59	Elution	1
59–69	Elution (membrane cleaning)	0

#### Spiking experiments

##### Spiking of ultrapure water with monodisperse NPs (80 and 150 nm) followed by microwave-assisted acidic digestion

All samples were directly prepared in the microwave quartz digestion tubes. Ultrapure water (1.8 mL) was spiked with 100 μL of the SiO_2_ stock suspensions (51,148 ppm for 150 nm and 10,480 ppm for 80 nm). To these diluted NP suspensions, 5 mL of 65% nitric acid and 1 mL of 30% hydrogen peroxide were added prior to the microwave-assisted digestion. After adding all aliquots, the samples remained at room temperature for 10 min for equilibration purposes. The samples were then digested under the following conditions: 5 min to reach 200 °C and held at 200 °C for 4 min. After cooling, the acidic samples were transferred from the microwave tubes to 100-mL volumetric flasks and filled to volume with the 0.25-mM ammonium carbonate solution. Exactly 3 mL of this suspension was then transferred to 50-mL Falcon™ tubes and brought to a volume of approximately 20 mL with the 0.25-mM ammonium carbonate solution. The pH of this suspension was adjusted to 9–9.5 with 1 M and 0.1 M sodium hydroxide solutions. Once the pH was set, the 0.25 mM ammonium carbonate solution was added to reach a final volume of 30 mL. Immersed in an ice bath, this suspension was probe sonicated (Sonics & Materials, Sonics VibraCell VCX-130, Inc., Newtown, CT, USA) using a 6-mm tip under various conditions (time, delivered acoustic power) with the aim of identifying the optimal sonication conditions (Table 3). The effective delivered acoustic power was determined following the approach described by Taurozzi and co-workers [20].

**Table 3:** Sonication conditions to which digested samples were exposed

Conditions	Duration [min]	Delivered acoustic power [W]	Delivered energy [J]
Condition 1	3	24	4320
Condition 2	7	24	10,080
Condition 3	12	24	17,280

Sonicated suspensions were then analysed by DLS and AF_4_, and the sizing results were compared with non-digested/non-sonicated (10 μL of stock solution in 100 mL of the 0.25 mM ammonium carbonate solution) and digested/non-sonicated suspensions at the same theoretical concentrations.

##### Spiking of ultrapure water with food-grade fumed silica (E551) followed by microwave-assisted acidic digestion

For the preparation of the stock solution, approximately 300 mg of food-grade synthetic amorphous silica (SAS), as described in the section “Food-grade synthetic amorphous silica E551 (SAS)”, was suspended in 25 mL of the 0.25 mM ammonium carbonate solution. This suspension was then vortex stirred for 10 s and probe sonicated for 10 min at approximately 24 W (total energy 14,400 J). Subsequently, the suspension was filtered through a 0.45-μm Millex-HV PVDF syringe filter (Merck Millipore, 25 mm, Product Code SLHV025NB). After the filtration step, the pH of the suspension was approximately pH 5–6. The pH was adjusted to pH 9–9.5. In the following step, the percentage of SiO_2_ in the stock solution that passed through the filter (recovery) was determined with ICP-MS and was found to be approximately 80%. To reach a concentration of 50 ppm (well visible in AF_4_-MALS), the above suspension required an overall dilution of 1:200. This was achieved by adding 1 mL of the stock solution to 1 mL of ultrapure water, 5 mL of 65% nitric acid and 1 mL of 30% hydrogen peroxide. The samples were digested under the conditions detailed in the section “Spiking of ultrapure water with monodisperse NPs (80 and 150 nm) followed by microwave-assisted acidic digestion”. After cooling, the acidic samples were transferred from the microwave tubes to 100-mL volumetric flasks and filled to volume with the 0.25 mM ammonium carbonate solution. Exactly 15 mL of this suspension was then transferred to 50-mL Falcon™ tubes and brought to a volume of approximately 25 mL with the 0.25 mM ammonium carbonate solution. The pH of this suspension was adjusted to 9–9.5 with 1 M and 0.1 M sodium hydroxide solutions. Once the pH was set, the 0.25 mM ammonium carbonate solution was added to reach a final volume of 30 mL. Immersed in an ice bath, this suspension was probe sonicated for 10 min at 24 W (total energy 14,400 J) using a 6-mm probe tip. Sonicated suspensions were then analysed by DLS and AF_4_, and the results compared to non-digested/non-sonicated (500 μL of stock solution in 100 mL of the 0.25 mM ammonium carbonate solution) and digested/non-sonicated suspensions at the same theoretical concentrations.

##### Spiking of tomato sauce with food-grade fumed silica (E551) followed by microwave-assisted acidic digestion

The spiking suspension was prepared following the procedure described in the section “Spiking of ultrapure water with food-grade fumed silica (E551) followed by microwave-assisted acidic digestion”. A defined amount (1 g) of tomato sauce was spiked with 1 mL of the E551 spiking solution. Subsequently, 5 mL of 65% nitric acid and 1 mL of 30% hydrogen peroxide were added. For the blank sample, instead of the spiked SiO_2_-NP suspension, 1 mL of ultrapure water was added. All other steps were as described in the section “Spiking of ultrapure water with food-grade fumed silica (E551) followed by microwave-assisted acidic digestion”.

## Results

### Impact of acidic microwave digestion on dissolution of particles and the release of ionic silicon

#### Optimization of the centrifugal filtration step

**Fig. 2:**
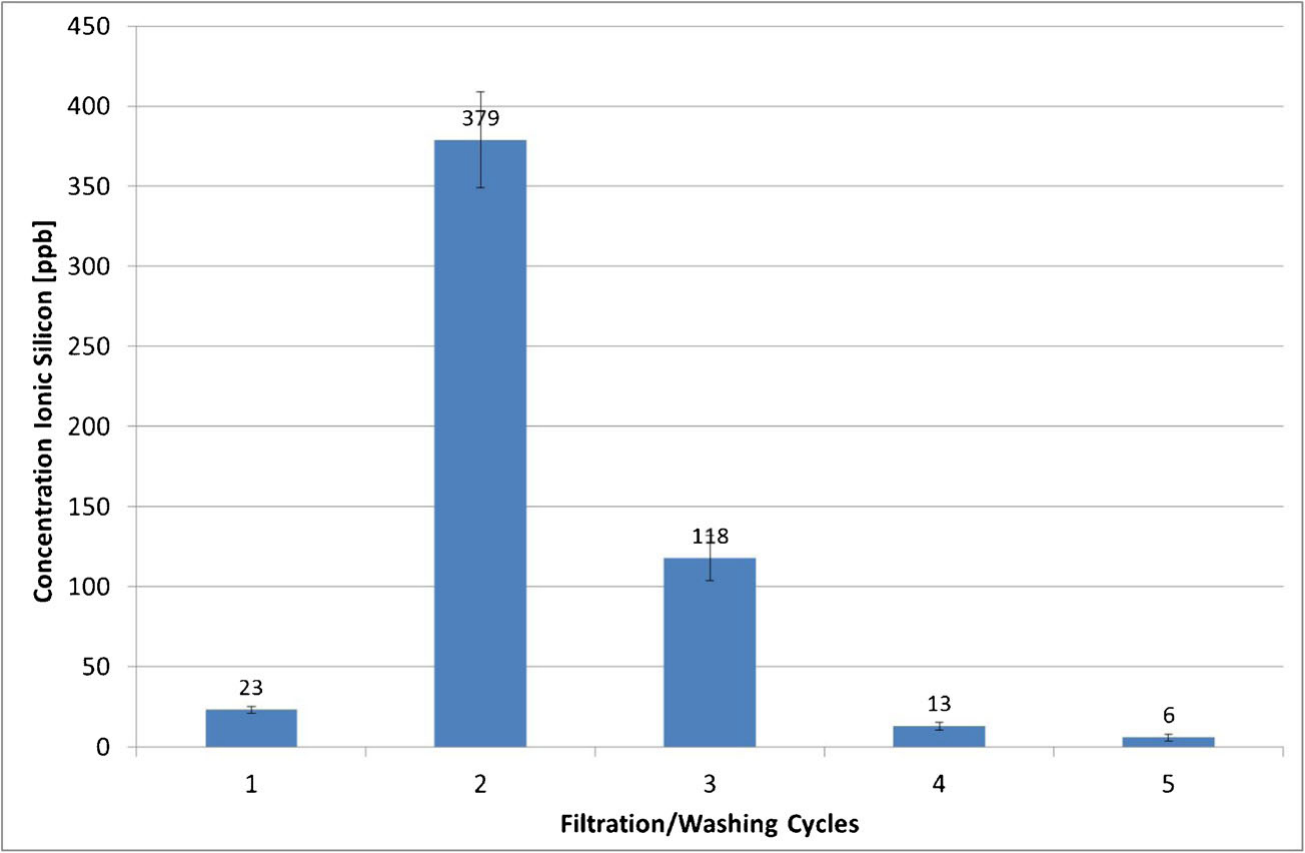
Ionic silicon released from Amicon® Ultra-4 centrifugal cellulose filter membranes after various washing cycles with ultrapure water (average concentrations ± SD, *n* = 3)

Figure 2 depicts the concentration of silicon in the filtrate after consecutive washing cycles. It appears that the cellulose filter membranes of the Amicon® Ultra-4 centrifugal filters released ionic silicon. The maximum release was reached with the second washing cycle (total volume of 8 mL ultrapure water). Afterwards, the release diminished, reaching 6 ppb in the filtrate after the 5th washing cycle. Considering that the ionic silicon content in the non-filtered ultrapure water was approximately 3 ppb, the concentration in the filtrate after the fifth washing cycle was considered low enough for the needs of this study.

In conclusion, for the assessment of ionic silicon release from silica particles, the centrifugal filters need to be washed for at least 5 washing cycles (of 4 mL each) before usage.

#### Retention efficacy of filtering membrane

The retention efficacy of the filtering membrane was assessed by filtering suspensions of SiO_2_ nanoparticle standards (20 to 150 nm), each at 200 ppb in 1% HNO_3_, and determining the concentration of ionic silicon in the filtrate. The results in Table 4 show that independent of the particle size, the concentration of ionic silicon measured in the filtrate was equal or close to that of the blank sample. Only the filtrate of the 80-nm particles showed a considerably higher concentration which does not, however, fit in any plausible particle size–ionic silicon concentration trend.

**Table 4:** Concentration of ionic silicon measured in the filtrate of nanoparticle suspensions of various sizes (average concentrations ± SD, *n* = 3)

Silica nanoparticle size [nm]	Average concentration of ionic silicon in the filtrate ± SD (*n* = 3) [ppb]
Blank (1% nitric acid)	22.0 ± 0.5
20 nm	21.6 ± 0.8
40 nm	22.6 ± 1.2
60 nm	23.5 ± 0.6
80 nm	32.2 ± 0.9
100 nm	22.8 ± 0.7
150 nm	21.1 ± 1.0

These results suggest that nanoparticles are fully retained independent of their size. This was an expected result, as the nominal molecular weight limit (or pore size) of the centrifugal filters used in this study was 3 kDa, which corresponds to a particle size of below 1 nm.

#### Microwave acidic digestion—optimization of conditions

Table 5 illustrates how milder digestion conditions (compared to those of the pre-set method) also lead to the complete digestion of the matrix. Digestion at 160 °C and a hold time of 4 min resulted in a completely clear digestion solution. It should, however, be noted that assessing the completeness of the digestion only by visual inspection might be associated with some uncertainty. Residual matrix components might interact with the particles in the sample and possibly interfere with their subsequent determination.

**Table 5:** Completeness of digestion in relation to digestion temperature and temperature hold time at constant ramp time (5 min)

Digestion temperature [°C]	Hold time at digestion temperature [min]	Visual evaluation of the sample [clear/not clear]
200	4	Clear
	3	Clear
	2	Clear
	1	Clear
180	4	Clear
	3	Clear
	2	Clear
	1	Clear
160	4	Clear
	3	Not clear
	2	Not clear
	1	Not clear
150	4	Not clear
	3	Not clear
	2	Not clear
	1	Not clear
120	1	Not clear
100	1	Not clear

These conditions were valid only for tomato sauce. The conditions might vary for different matrices.

#### Correlation of SiO_2_-NP size and dissolution propensity under acidic digestion conditions (two target temperatures—160 °C and 200 °C)

The aim of these measurements was to investigate the potential relationship between particle size and dissolution propensity during the digestion procedure at two different digestion temperatures (160 °C and 200 °C). The dissolved fraction was calculated by subtracting the mass concentration of Si-28 after filtration from that before filtration. The results are expressed as a percentage according to formula (3):

3 $$ \mathrm{Dissolved}\ {\mathrm{Si}\mathrm{O}}_2\ \left[\%\right]=\left(\mathrm{mass}\ {\mathrm{concentration}}_{\mathrm{Si},\mathrm{ionic}}\ \left[\mathrm{ppb}\right]/{\mathrm{mass}\ \mathrm{concentration}}_{\mathrm{Si},\mathrm{total}}\ \left[\mathrm{ppb}\right]\right)\ast 100 $$

where (mass concentration_Si,ionic_) is the Si-28 mass concentration in the filtrate determined after ultracentrifugation through Amicon filters, and (mass concentration _Si,total_) is the Si-28 mass concentration in the digested and diluted mixture before ultracentrifugation. The concentration of silicon determined in the blank sample was subtracted from all test samples. Recovery of the 200-ppb silicon control sample was 96.8%, indicating that no major losses occurred during the filtration step.

**Fig. 3:**
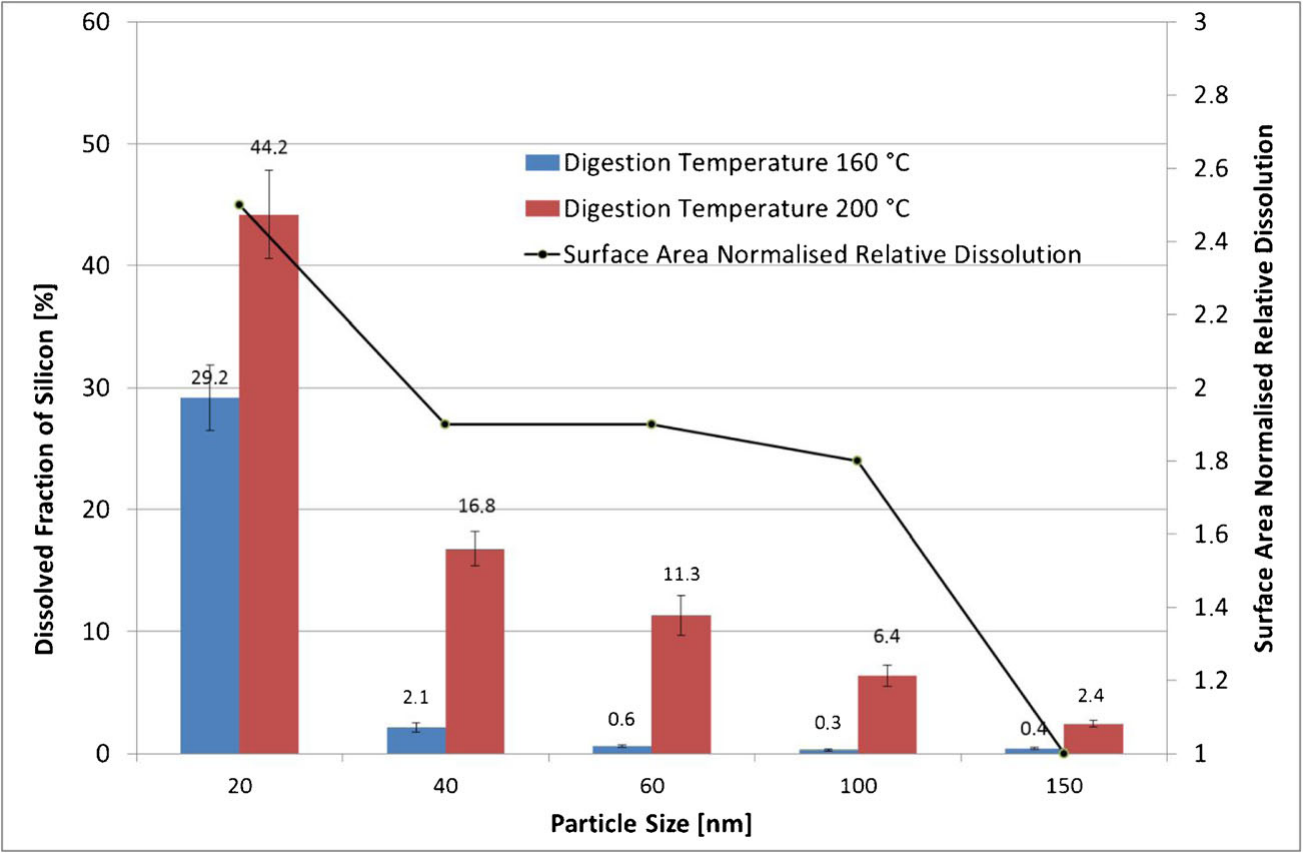
Effect of digestion temperature and particle size on SiO_2_ dissolution. Left *y*-axis: average dissolved fraction of silicon in percent ± SD (*n* = 3); right *y*-axis: surface area normalized relative dissolution (only for 200 °C digestion temperature)

Figure 3 shows that particles of all sizes and at both digestion temperatures underwent dissolution. A clear trend can be observed: the digested suspension which contained smaller particles dissolved to a higher extent compared to the one with larger particles. The highest dissolution rate (44%) was observed for particles with a nominal size of 20 nm at a digestion temperature of 200 °C. Dissolution depends on the materials’ solubility within a given environment as well as the concentration gradient between the particle surface and the bulk solution phase. Consequently, the kinetics of dissolution of soluble materials is surface area dependent. Particles of a smaller size dissolve faster and to a higher extent than larger materials of the same mass [21–23]. The overall surface area of the 20-nm particle suspension is 7.5 times higher than that of the 150-nm particles. The line chart in Fig. 3 depicts the surface area normalized relative dissolution at a digestion temperature of 200 °C. These values were obtained dividing the dissolved fractions, expressed in percent, by the relative surface areas of each particle size, at the same mass. These values were then again normalized, assigning an arbitrary unit of 1 to the largest particles (150 nm). The other values express how many times more ionic silicon is dissolved compared to the 150-nm particles. Results show that the surface area normalized dissolution of the 20-nm particles is approximately 2.5 times higher than that of the 150-nm particles. Particles with a size of 40, 60 and 100 nm show a constant surface area normalized relative dissolution of 1.8–1.9 times that determined for the 150-nm particles. A possible explanation for this is the higher propensity of smaller particles to dissolve due to their energetically unfavourable higher surface-to-volume ratio [21, 24]. This finding is confirmed by Roelofs and co-workers [25] who investigated the dissolution of various types of synthetic amorphous silica.

The second important finding is that a lower digestion temperature also resulted in a lower dissolution percentage. The temperature of 160 °C was chosen since this is the minimum temperature required to achieve a full digestion of tomato soup. The dissolution rates at 160 °C were negligible for particles with a nominal size of > 60 nm, while for smaller particles, the dissolution was significant (almost 30% for 20-nm particles). Giovannini et al. [26] studied the dissolution of microporous silica nanoparticles in aqueous environments at different biologically relevant pH values to assess their potential as a drug delivery vehicle. The authors found that nanoparticles degraded at pH 6 and pH 7.4, while no degradation was found at pH 4. Under alkaline conditions, silanol groups undergo a stronger deprotonation, and the hydrolysis of Si–O–Si bonds is catalysed by nucleophilic attack of the OH^−^ ions. In addition, Braun and co-authors [27] determined the influence of the composition of different simulated body fluids on the observed silica dissolution rates and found similar results. The dissolution rates were found to be higher at a pH of approximately 7, whereas at pH 1.6, the dissolutions were low. These discrepancies with our findings can be explained by the conditions under which the dissolution tests were conducted. The conditions used during the microwave-assisted digestion were far more aggressive than those of the two studies described above. Moreover, during the digestion procedure in our experiments, the pH values cross the isoelectric point at which the particles are free of charge and tend to agglomerate/aggregate, which might also have an impact on the dissolution properties of the particles.

#### Solubility of polydisperse food-grade (E551) fumed silica under acidic digestion

In the preceding section, the dissolution propensity of well-defined monodisperse silica nanoparticles was discussed. This part, in contrast, aims at investigating the dissolution of polydisperse food-grade silica. First, a suspension of 1000 ppm was filtered through a 0.45-μm PTFE disc filter. A 100-μL aliquot of the filtrate was then diluted with 1% nitric acid and analysed with ICP-MS. The filtrate contained 547 ppb silicon. In the next step, the 547-ppb suspension was ultracentrifuged through 3-kDa Amicon filters. The filtrate contained 22 ppb of silicon, which corresponded to a 4% portion of the ionic silicon in the investigated E551 fumed food-grade silica when suspended in 1% HNO_3_. This finding is in good agreement with the findings of Aureli and co-authors [17], who found that ionic and particulate fractions always coexist in nanosilica (undigested) polydisperse suspensions and quantified the ionic part of various polydisperse silica suspensions in the range of 0.5–15%, depending on the type of material.

For the assessment of the impact of acidic digestion on the amount of dissolved silicon, 100 μL of the disc-filtered filtrate was transferred into a microwave digestion vessel along with 5 mL of concentrated nitric acid, 1 mL of 30% hydrogen peroxide and 1.9 mL ultrapure water. The microwave-digested suspension was then diluted to 1:1000 with 1% nitric acid and analysed with ICP-MS. The concentration of silicon was found to be 480 ppb. The missing 67 ppb might have remained attached to the walls of the extraction vessel. The ultracentrifuged filtrate of the microwave-digested suspension contained 98 ppb silicon, corresponding to a 20% portion of the dissolved silicon from the food-grade fumed silica. These results (2 replicates, *n* = 3) demonstrate that during the microwave-assisted acidic digestion, a part—in particular, the smaller ones—of the particles dissolve. This action has an impact on the number-size distribution of particles.

### Impact of acidic digestion on the particle size distribution

#### Spiking experiments

##### Spiking of ultrapure water with monodisperse NPs (80 and 150 nm) followed by microwave-assisted acidic digestion

**Fig. 4:**
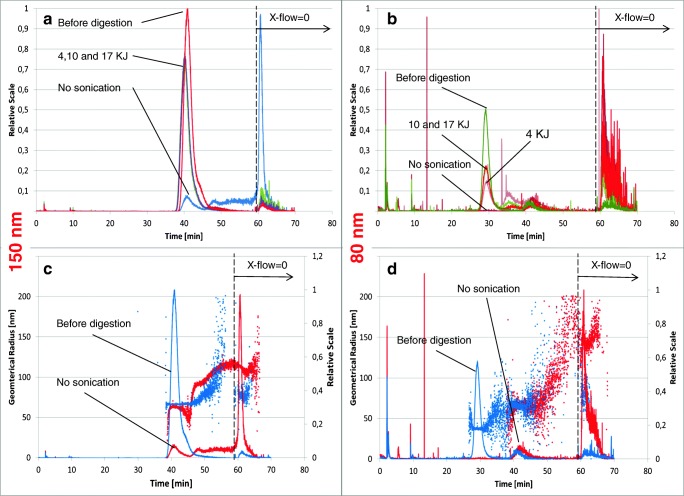
**a**–**d** Upper part: overlaid AF_4_-fractograms (MALS) of non-digested silica NP suspensions, digested but not sonicated suspensions and digested suspensions sonicated with various amounts of energy (150 nm [**a**] and 80 nm [**b**] particles). Lower part: overlaid fractograms of 150 nm and 80 nm SiO_2_-NP non-digested samples and digested but not sonicated samples and their respective geometric radii (nm) recorded with the multi-angle laser light scattering (MALS) detector

Figure 4a shows the overlaid fractograms of the non-digested 150-nm nanoparticle suspension (red line), the digested but not sonicated suspension (blue line) and the digested suspensions exposed to various amounts of sonication energy (green, pink and brown lines).

After digestion and before sonication, the vast majority of particles that originally had a size of 150 nm were no longer visible in the fractogram. Instead, a large peak appeared when excluding the cross-flow at the end of the method for the removal of large particles left on the membrane surface of the AF_4_ channel. In Fig. 4c, the geometric radii recorded with the multi-angle laser light scattering (MALS) detector are displayed for both the not-digested (blue line) and the digested but not sonicated NP suspensions (red line). The particle size distribution of the digested but not sonicated sample was no longer monodisperse; the size of the eluted particles steadily increased starting from the retention time of the main peak (at approximately 40 min) to the end of the elution before the cross-flow was set to zero. The initial size of the particles (approximately 65 nm, geometric radius) increased continuously until a plateau at approximately 120 nm was reached. The fact that the dominant peak appearing just after 60 min (no cross-flow) did not provide a distinct size measurement could indicate that only a small amount of very large particles were eluted at that retention time.

Figure 4a shows that sonication of the digested samples, the pH of which had previously been adjusted to approximately 9–9.5, partially re-establishes the original particle size distribution. A similar impact of the digestion and sonication can also be observed for the 80-nm SiO_2_ nanoparticles (Fig. 4b). However, the amount of sonication energy seems to have a more direct and proportional impact on the efficiency of re-establishing the original particle size distribution. The higher the sonication energy is, the closer the size distribution approaches the original non-digested suspension. It is interesting to see that a second particle population appeared at a retention time of approximately 35 min, which decreased as the digested sample became more sonicated. The average geometric radius of this second population was approximately 65 nm compared to the 40 nm of the non-digested particles (Fig. 4d).

**Fig. 5:**
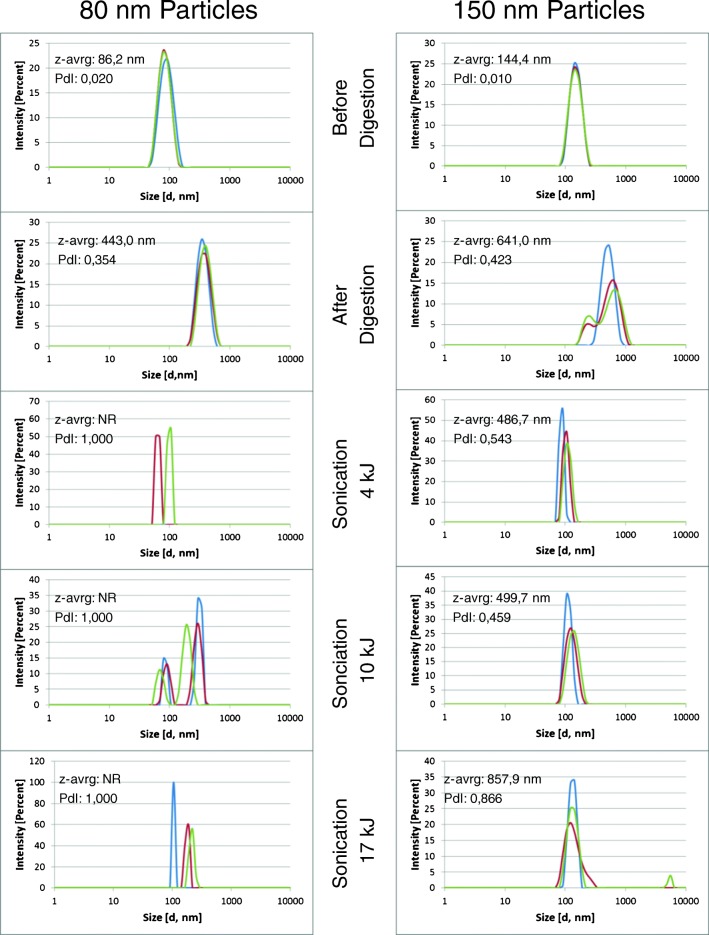
DLS measurements (size distribution by intensity) for both 80-nm (left column) and 150-nm (right column) nanoparticles at various stages of the digestion procedure and at increasing sonication energy doses (each spectrum includes 3 replicate measurements). NR = no result

The general increase in the particle size after digestion and the incomplete recovery of the initial size distribution can also be observed from the DLS spectra (Fig. 5) recorded with the same procedural steps that the AF_4_ fractograms were acquired. Although providing less detail, the results obtained from the DLS analysis confirm that the original particle size distribution was not reached again, independent of the amount of energy that the digested suspension was exposed to. Generally, it appears that the original particle size distribution was more easily re-established by digesting and sonicating the 150-nm monodisperse nanoparticles than the 80-nm nanoparticles. After the digestion and sonication of the 80-nm nanoparticles, a number of secondary- and tertiary-size populations appeared that did not entirely disappear, even when exposed to high sonication doses.

The reason for the increase in the average particle size and the shift in the particle size distribution can be attributed to the aggregation/agglomeration behaviour of silica suspensions [4, 28]. Amorphous silica surfaces contain silanol groups. The charge of these groups determines the repulsive properties among silica particles [29], and due to protonation/deprotonation of the silanol groups, the charge depends on the pH of the medium in which the particles are dispersed [28]. The isoelectric point at which the particles carry no net charge, and where the aggregation is therefore the strongest, is at pH 2–3. Peters and co-authors [4] found in their work that large silica agglomerates formed at low pH values and deagglomerated again at pH values above 7.

The issue of SiO_2_ nanoparticle instability/agglomeration was also observed by Grombe and co-authors [8] during their efforts to produce reference materials for the detection and size determination of silica nanoparticles in tomato sauce. According to their findings, after the removal of organic material by acidic digestion, the stabilization of the remaining particle suspension by adjusting the pH and probe sonication, the initial particle size distribution could be partially re-established.

##### Characterization of food-grade polydisperse fumed silica (E551)

**Fig. 6:**
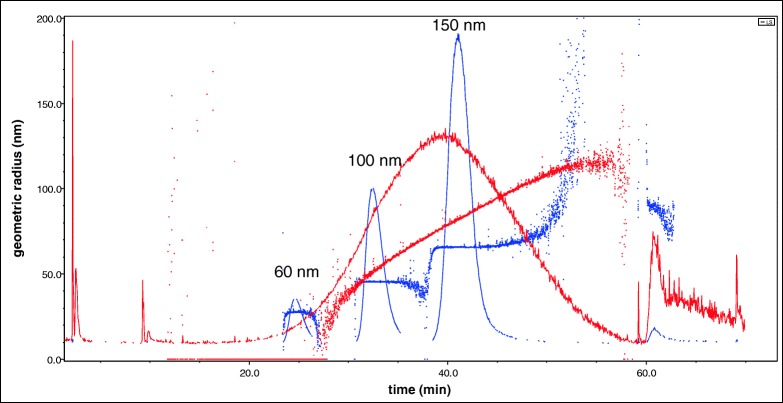
Fractogram of 20-ppm filtered suspension of food-grade fumed silica overlaid with 10 ppm monodisperse size standards (60 nm, 100 nm and 150 nm). The light scattering signal was recorded with MALS at 90°. On the left *y*-axis, the geometrical radius is displayed. The signals are normalized

A 20-ppm filtered (0.45 μm) suspension of the food-grade fumed silica used in this study (red line) was injected into the AF_4_-MALS system together with a mixture of (10 ppm each) monodisperse 60 nm, 100 nm and 150 nm SiO_2_-NP size-standard suspensions (blue line). The fractogram in Fig. 6 shows that a relevant number of particles were in the size range below 100 nm. The scattering intensity signal recorded with the 90° MALS (red line) exhibits an almost perfectly symmetrical shape with a peak apex just below 150 nm. However, considering that the scattering intensity is proportional to the particle diameter to a power of 6, the majority of particles (in number) have a size smaller than 150 nm.

**Fig. 7:**
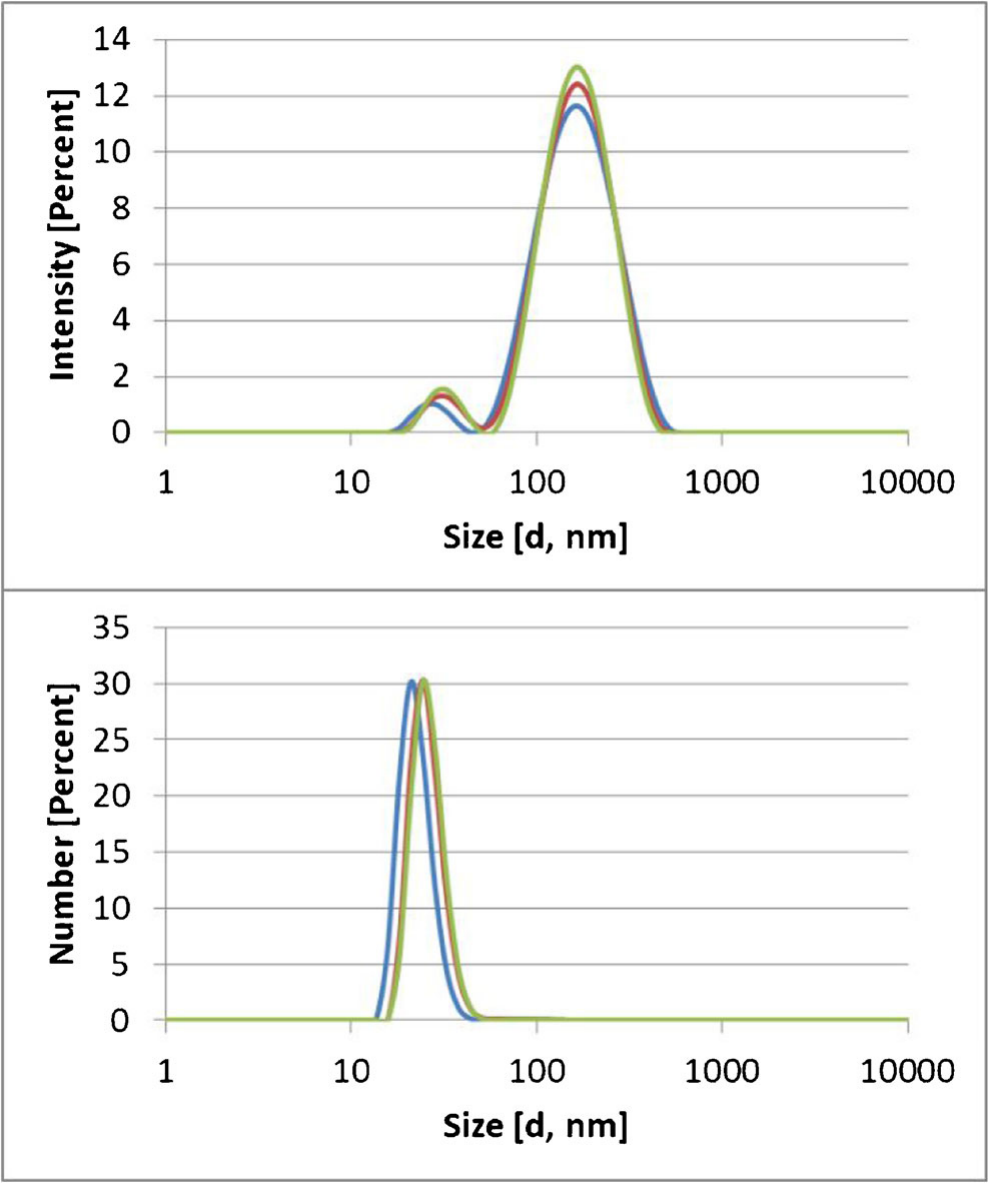
DLS spectra of sonicated and filtered E551. In the upper part, the relative scattering intensity is chosen as the unit of the *y*-axis. In the lower part, the extrapolated relative number of particles is chosen as unit of the *y*-axis

This result is also supported by the DLS spectra in Fig. 7 in which both the raw scattering intensities and the extrapolated number percentages are shown for the sonicated and filtered E551 suspension. The *z*-average was 133.4 nm, and the polydispersity index (PdI) was 0.238. However, most particles were in the size range between 20 and 30 nm.

##### Spiking of ultrapure water with food-grade fumed silica (E551) followed by microwave-assisted acidic digestion

**Fig. 8:**
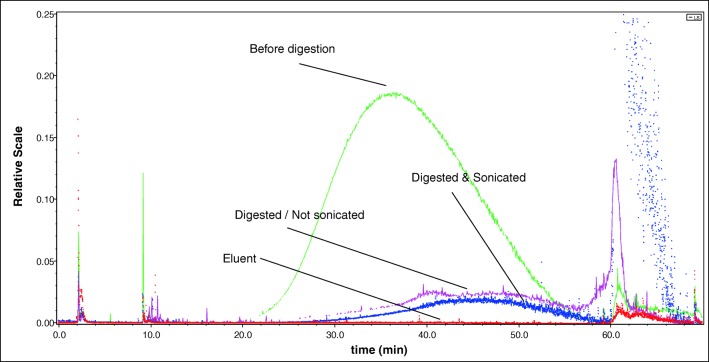
Fractogram (AF_4_-MALLS, LS angle 90°) of the E551 sample before digestion, digested but not sonicated, digested and sonicated and the eluent (0.25 mM ammonium carbonate)

After the digestion, hard-to-remove precipitates on the walls of the digestion tubes became visible (see Electronic Supplementary Material (ESM) Fig. S1). This might be one of the reasons for the low overall recovery (approx. 25%), which can be observed in the fractogram depicted in Fig. 8 (blue and pink lines compared to green line). The recovery rate of the digested/sonicated and digested/non-sonicated samples was determined by setting their integrated peak areas, recorded with the LS 90° light scattering signal, in the elution time window starting at 16 min and ending at 59 min, in relation to the peak area of the undigested sample in the same time window.

No differences in recovery were observed between the probe-sonicated digested suspension (blue line) and the non-sonicated digested (pink line) sample. When comparing the fractograms obtained from the analysis of the non-digested and the digested/sonicated suspension, it can be observed that in the digested suspension the peak-apex has shifted to the right, meaning that the suspension after digestion/sonication contained generally larger particles.

##### Spiking of tomato sauce with food-grade fumed silica (E551) followed by microwave-assisted acidic digestion

**Fig. 9:**
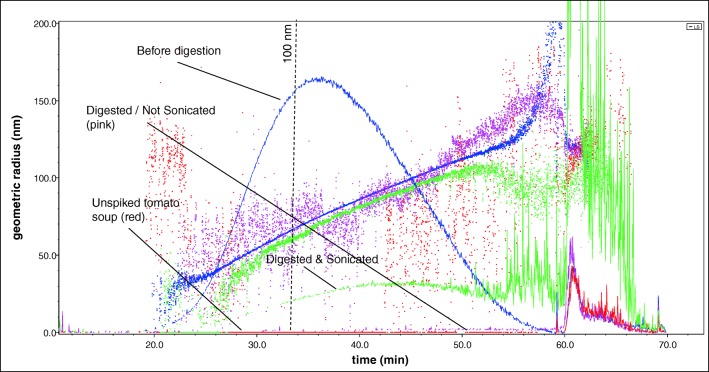
Fractogram (AF_4_-MALS, LS angle 90°) of tomato sauce spiked with E551 before digestion, digested but not sonicated, digested and sonicated and the blank non-spiked tomato sauce

The microwave-assisted digestion was able to remove all organic matrix components. When adding nitric acid and hydrogen peroxide without heating, a large portion of the matrix already decomposed, resulting in a yellow, not completely clear, extract. After the microwave-assisted digestion, the extracts were clear and a yellowish colour (see ESM Fig. S2). Compared to the spiked ultrapure water, the digested but not sonicated sample (red line in Fig. 9) only showed a very weak signal in the retention time range at which the particles of the E551 should be visible. The signal re-appeared, however, after probe sonication (green line). The recovery rate of the digested/sonicated sample was determined as described under section “Spiking of ultrapure water with food-grade fumed silica (E551) followed by microwave-assisted acidic digestion” and was 28.1 ± 8.1% (3 replicates). In a previous study conducted by Tadjiki and co-workers [30], the recoveries of SiO_2_ nanoparticles were found to be in a similar range (25–39%). In another study, in which SiO_2_ nanoparticles were extracted from tomato sauce, the recovery was, however, substantially higher, > 90% [31].

In all three replicates, an elution shift towards later elution times was observed, meaning that the number-size distribution shifted towards larger particles. The extent of this shift varied slightly between the three replicates, indicating that the deagglomeration did not occur in the same way for all replicates. The slight shift in size distribution confirms the findings of Wagner and co-authors [31]. In their study, they found that despite careful adjustment of the particle stabilization conditions, a slight shift was inevitable. Peters et al. [4] reached the same conclusion in their study of the dissolution, agglomeration and release of materials in the nanosize range from food with E551 during human digestion.

## Conclusions

The results of this study demonstrate that microwave-assisted acidic digestion partially dissolves silica nanoparticles. The digestion conditions (pH), moreover, lead to a strong agglomeration of the particles. A complete deagglomeration is not achieved, even when exposed to elevated sonication doses. The consequence of these two findings is that the size distribution of the particles after acidic digestion differs from the original distribution before digestion. Microwave-assisted acidic digestion is therefore unsuitable for the removal of organic matrices when the final objective is to evaluate whether the tested material is a nanomaterial or not, according to the current definition recommended by the European Commission [32]. A milder process of matrix elimination, such as enzymatic or UV treatment, should be preferred when the unaltered number-size distribution is required.

## Electronic supplementary material

ESM 1 (PDF 361 kb)
