# Supplementary material for: Challenges in isolating silica particles from organic food matrices with microwave-assisted acidic digestion
Source: Anal Bioanal Chem. 2019 Jun 21;411(22):5817–31. doi: 10.1007/s00216-019-01964-2 (PMC6704109; doi:10.1007/s00216-019-01964-2)
Supplement: Supplementary file 1 — (PDF 361 kb) [file 216_2019_1964_MOESM1_ESM.pdf]

Analytical and Bioanalytical Chemistry

Electronic Supplementary Material

Challenges in isolating silica particles from organic food matrices with microwave-assisted acidic digestion

Otmar Geiss, Ivana Bianchi, Chiara Senaldi, Josefa Barrero

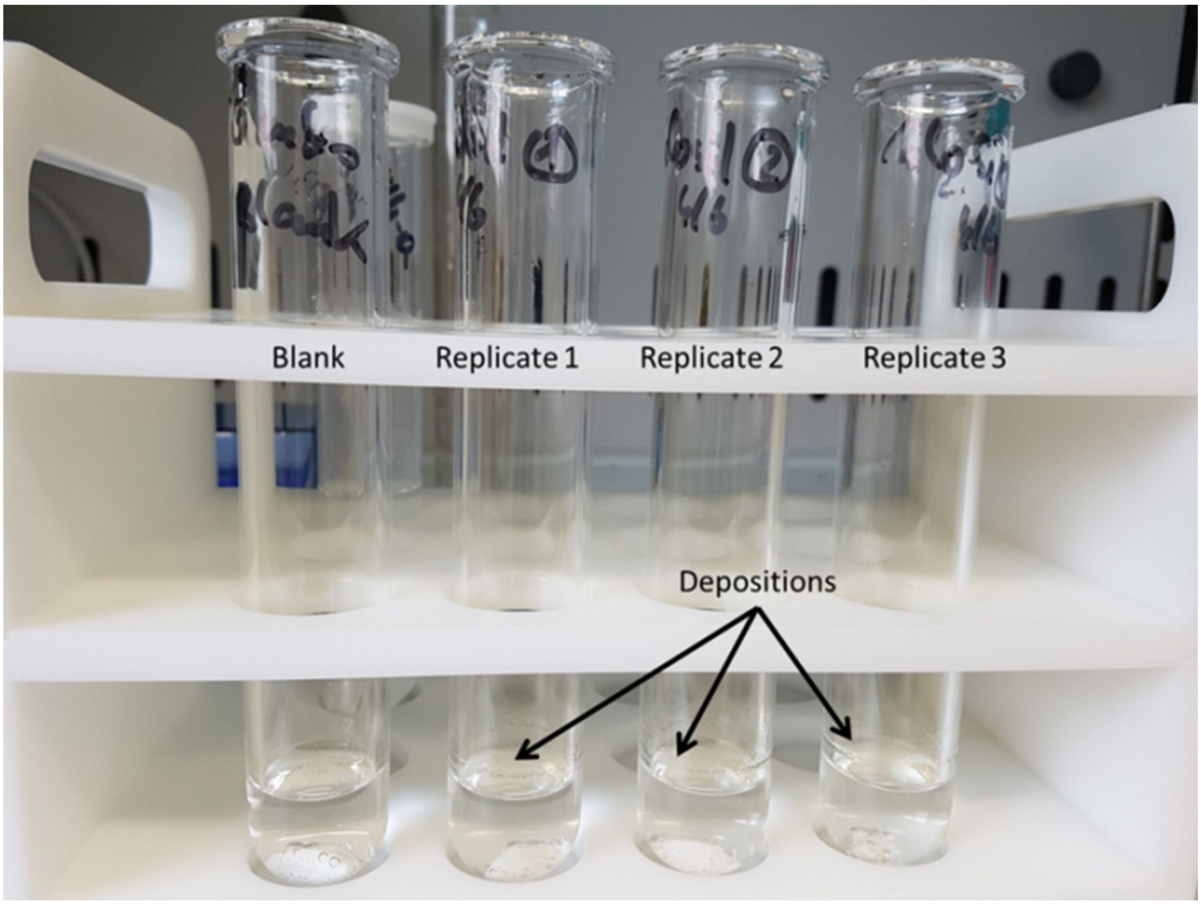

Fig. S1 Hard to remove precipitates on the digestion tubes becoming visible after digestion

Appearance of tomato sauce at different stages of the digestion process

The microwave assisted digestion was able to remove all organic matrix components. Already when adding nitric acid and hydrogen period without heating, most of the matrix is decomposed resulting in a yellow, not completely clear extract. After the microwave assisted digestion, the extracts were clear and of yellowish colour.

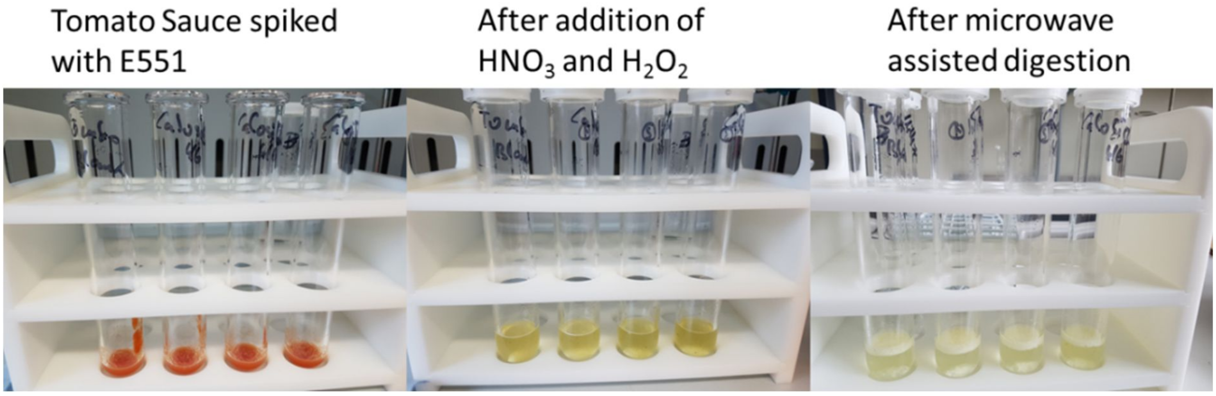

Fig. S2 The spiked tomato sauce at three different stages. Left: before adding acid and hydrogen peroxide, centre: after adding acid and hydrogen peroxide but before microwave, right: after microwave assisted digestion
